# Incidence of Cancer in Adolescents and Young Adults in Jordan, 2000-2017

**DOI:** 10.1200/GO.21.00007

**Published:** 2021-06-17

**Authors:** Justin Z. Amarin, Razan Mansour, Omar F. Nimri, Maysa Al-Hussaini

**Affiliations:** ^1^ Office of Scientific Affairs and Research, King Hussein Cancer Center, Amman, Jordan; ^2^ Jordan Cancer Registry, Ministry of Health, Amman, Jordan; ^3^ Department of Pathology and Laboratory Medicine, King Hussein Cancer Center, Amman, Jordan

## Abstract

The epidemiology of cancer in adolescents and young adults (AYAs) is distinct and underinvestigated. Therefore, we investigated the incidence of cancer in AYAs in Jordan.

**PATIENTS AND METHODS:**

We accessed all records submitted to the Jordan Cancer Registry between 2000 and 2017. We included all patients, age 15-39 years, who were ordinarily resident in Jordan. We then calculated frequencies, age-adjusted incidence rates (AAIRs), and annual percentage changes (APCs) and performed subgroup analyses by biologic sex, age subgroups, and site (SEER AYA site recode/WHO 2008). We also performed site-specific trend analyses using joinpoint models.

**RESULTS:**

We identified 14,115 eligible patients, of whom 1,531 (10.8%), 4,278 (30.3%), and 8,306 (58.8%) were 15-19, 20-29, and 30-39 years old at diagnosis, respectively. The numbers of male and female AYAs were 5,792 (41.0%) and 8,323 (59.0%), respectively. The crude number of cases increased from 654 in 2000 to 954 in 2017 (APC, 2.6%). The overall AAIR ranged from 32.3 in 2000 to 24.3 in 2017 (APC, –1.7%). The AAIR was 27.6 over the full study period and was higher in females (34.1) than in males (21.6). Carcinomas, lymphomas, and leukemias were the most common cancers. The incidence rates of the majority of cancers trended downward over the study period.

**CONCLUSION:**

The incidence of cancer in AYAs in Jordan is relatively low and declining. However, the absolute number of cases is increasing because this downtrend does not offset the effect of a high population growth rate; almost a 1,000 cases of cancer are now diagnosed every year, which represents a significant increase in the burden of cancer in a developing country with limited healthcare resources.

## INTRODUCTION

The National Cancer Institute's Adolescent and Young Adult Oncology Progress Review Group defines adolescents and young adults (AYAs) with cancer as those diagnosed at age 15-39 years.^1^ AYAs are distinct from children and older adults because of important differences among the three population groups in the distribution of cancer sites, risk factor profiles, host and disease biology, survivorship, and long-term health consequences.^2^ These differences are clinically significant because, historically, advances in cancer therapy have benefitted children and older adults more so than AYAs.^3^ In addition, the burden of cancer in AYAs is generally increasing; Gupta et al^4^ studied AYAs with cancer in 41 countries over a 15-year period (1998-2012) and found that the incidence increased in 23 countries, decreased in two, and remained the same in 16. According to the GLOBOCAN 2018 estimates, cancer was diagnosed in 1.2 million AYAs worldwide.^5^

**CONTEXT**

**Key Objective**
The epidemiology of cancer in adolescents and young adults (AYAs) in Jordan has not been investigated. AYA oncology is particularly relevant in developing countries because AYAs comprise a larger proportion of the population. Our aim was to describe the incidence of cancer in AYAs in Jordan.
**Knowledge Generated**
We accessed data from a population-based registry and described the incidence of cancer in 14,115 AYAs in Jordan (2000-2017). Although the crude number of cases generally increased annually, the age-adjusted incidence rate generally decreased. Carcinomas, lymphomas, and leukemias were the most common cancers. The overall incidence of cancer was higher in female AYAs, largely because of the higher incidence of thyroid and breast carcinomas.
**Relevance**
Because of the high population growth rate, the absolute number of cases is increasing despite the downtrend in cancer incidence. Therefore, the burden of cancer in AYAs is increasing in a developing country with limited healthcare resources.

**FIG 1:**
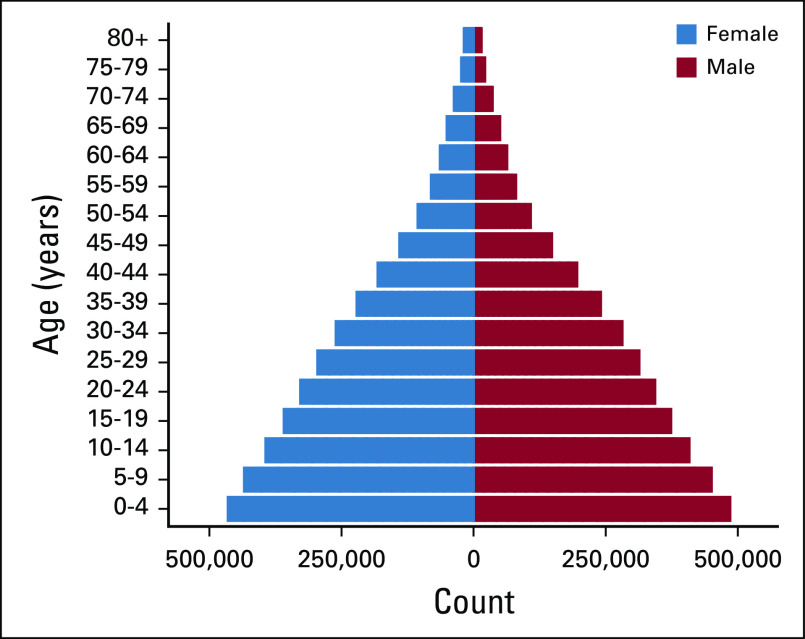
Pyramid plot of the mean population estimates in Jordan by age group and sex (2000-2017). Reproduced with permission.^11^

Cancer statistics for AYAs are often presented in aggregate with those of children or older adults, which obfuscates the distinct epidemiology of cancer in this population group. In addition, classification schemes that are not tailored to AYAs poorly capture the major cancer sites that affect these individuals.^6^ Many studies have documented the incidence of cancer in Jordan using data from the Jordan Cancer Registry (JCR), a high-quality population-based cancer registry.^7-10^ However, none has presented aggregate data for AYAs, and none has used a classification scheme tailored to AYAs. Jordan is a developing country with a rapidly growing population characterized by an expansive population pyramid (Fig 1). Therefore, this research gap is particularly pressing given that AYAs in Jordan comprise a larger proportion of the total population compared with AYAs in developed countries. For example, the mean proportion of AYAs between 2000 and 2017 was 42.7% in Jordan and 34.4% in the United States.^11^ To address the research gap, we accessed data from the JCR and described the incidence of cancer in AYAs in Jordan (2000-2017) using the SEER AYA site recode/WHO 2008.

## PATIENTS AND METHODS

We accessed all records submitted to the JCR between January 2000 and December 2017. The JCR, a population-based registry established in 1996, is included in the Cancer Incidence in Five Continents Volume XI database. The registry collects data from all hospitals and laboratories in Jordan using passive and active methods (eg, notification forms and case finding, respectively), and reporting is mandatory. The JCR is a member of a network of population-based registries sponsored by the Middle East Cancer Consortium (MECC). The MECC was established in 1996 with the support of the United States National Cancer Institute; University of California, Irvine; Emory University; and the International Agency for Research on Cancer.^12,13^ The immediate aim of the MECC was to establish the network and ensure the comparability of data.^12^ The network operates according to the standards outlined in the Manual of Standards for Cancer Registration (first disseminated in December 1998).^14^ The steering committee of the MECC routinely performs external audits to assure data consistency and quality. According to these audits, the coverage rate of the JCR falls between 92% and 97%.^13^

We filtered the records to include all patients, age 15-39 years, who were ordinarily resident in Jordan. We interrogated the World Development Indicators database to retrieve population estimates. The estimates include all residents regardless of legal status or citizenship.^11^ We then reformatted the collated data using SEER*Prep (version 2.5.8) and calculated frequencies, age-adjusted incidence rates (AAIRs), and annual percentage changes (APCs) using SEER*Stat (version 8.3.8). The reference population was the World Standard Population. We also performed subgroup analyses by biologic sex, age subgroups (15-19, 20-29, and 30-39 years), and site (SEER AYA site recode/WHO 2008). Finally, we used Joinpoint Regression Program (version 4.8.0.1) to perform site-specific trend analyses using joinpoint models. We calculated 95% CIs using the method described by Tiwari et al^15^ as implemented in SEER*Stat, and we interpreted values of *P* ≤ .05 to indicate statistical significance.

## RESULTS

We examined 20,318 patients for eligibility and included 14,115 (69.5%) who were ordinarily resident in Jordan. Of the patients we included, 1,531 (10.8%) were 15-19 years old at diagnosis, 4,278 (30.3%) were 20-29 years old at diagnosis, and 8,306 (58.8%) were 30-39 years old at diagnosis. The median age at diagnosis was 31 years (IQR, 25-36 years). The number of female AYAs was 8,323 (59.0%) compared with 5,792 male AYAs (41.0%), for a male-to-female ratio of 1:1.4. A small majority of patients were residents of the Amman Governorate (n = 7,917 [56.1%]), and the majority were Jordanian nationals (n = 13,874 [98.3%]). In 2017, the proportion of Jordanian nationals was 99.1%.

**FIG 2:**
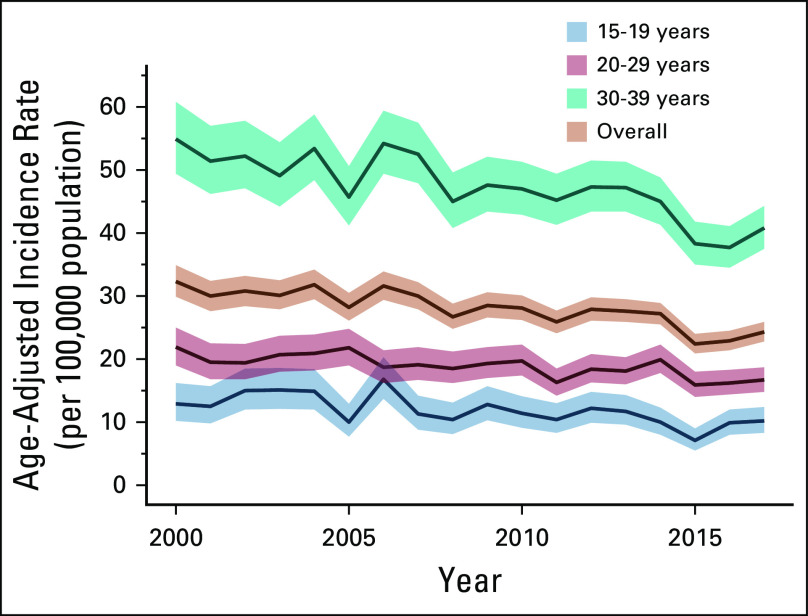
Line plots of the annual age-adjusted incidence rates of all cancers diagnosed in adolescents and young adults (15-39 years) in Jordan, 2000-2017 (N = 14,115). Data are stratified by the 15-19, 20-29, and 30-39 age subgroups. Error bands are 95% CIs.

The crude number of cases increased from 654 in 2000 to 954 in 2017. The AAIRs were 27.6 (27.1 to 28.0 years) overall, 11.6 (11.0 to 12.2 years) for those age 15-19 years, 18.7 (18.1 to 19.2 years) for those age 20-29 years, and 46.5 (45.5 to 47.5 years) for those age 30-39 years. The overall AAIR ranged from 32.3 (29.9 to 34.9) in 2000 to 24.3 (22.8 to 25.9) in 2017 for a total APC of –1.7% (–2.3 to –1.2; *P* < .001). The trends of the 15-19, 20-29, and 30-39 age subgroups were similar to the overall trend (Fig 2). The total APC in the AAIRs was –2.5% (–4.0 to –1.1; *P* = .002), –1.4% (–2.0 to –0.7; *P* < .001), and –1.8% (–2.4 to –1.1; *P* < .001) for those age 15-19 years, 20-29 years, and 30-39 years, respectively.

The overall AAIR was higher in female AYAs (34.1 [33.4 to 34.8]) than in male AYAs (21.6 [21.0 to 22.1]). The rates did not differ between females and males in the 15-19 age subgroup (11.0 [10.2 to 11.9] and 12.2 [11.4 to 13.0], respectively). However, in the 20-29 age subgroup, the rates differed between females and males (20.8 [20.0 to 21.7] and 16.7 [16.0 to 17.4], respectively). In addition, the rates of females and males in the 30-39 age subgroup (61.8 [60.2 to 63.5] and 32.3 [31.2 to 33.5], respectively) differed, and this rate difference was the main contributor to the overall rate difference.

We classified cases according to the SEER AYA site recode/WHO 2008 and calculated the crude number of cases, proportion of cases, and AAIRs accordingly (Table 1 and Table A1). We also calculated the female-to-male AAIR ratios (Table A2). The most common cancers in the 15-19 age subgroup were lymphomas (n = 491 [32.1%]; AAIR = 3.7), leukemias (n = 271 [17.7%]; AAIR = 2.1), and carcinomas (n = 246 [16.1%]; AAIR = 1.9). The most common lymphoma and leukemia were Hodgkin lymphoma (71.9%) and acute lymphoid leukemia (54.6%), respectively. Hodgkin lymphoma disproportionately affected female AYAs (AAIR ratio = 0.58 [0.42 to 0.79]; *P* < .001), whereas acute lymphoid leukemia disproportionately affected male AYAs (AAIR ratio = 1.53 [1.23 to 1.90]; *P* < .001). Carcinomas of the head and neck, including thyroid carcinoma, accounted for 67.5% of all carcinomas. Thyroid carcinoma was disproportionately incident in female AYAs (AAIR ratio = 5.84 [3.21 to 11.49]; *P* < .001). During the 18-year study period, one case of breast carcinoma was diagnosed in this age subgroup (0.1%).

**TABLE 1:**
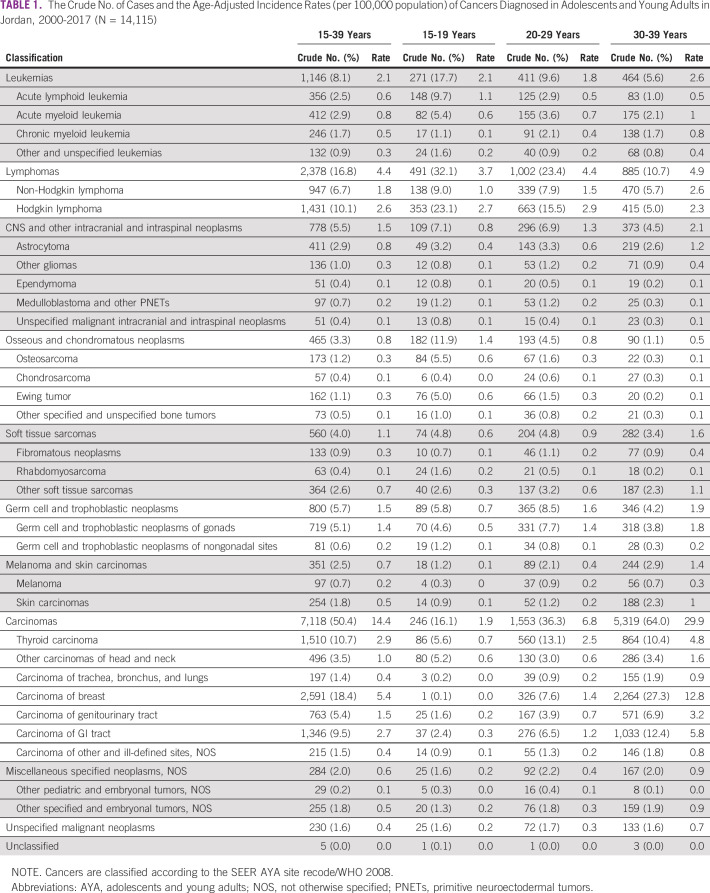
The Crude No. of Cases and the Age-Adjusted Incidence Rates (per 100,000 population) of Cancers Diagnosed in Adolescents and Young Adults in Jordan, 2000-2017 (N = 14,115)

In the 20-29 age subgroup, the most common cancers were carcinomas (n = 1,553 [36.3%]; AAIR = 6.8), lymphomas (n = 1,002 [23.4%]; AAIR = 4.4), and leukemias (n = 411 [9.6%]; AAIR = 1.8). The most common lymphoma was, again, Hodgkin lymphoma (66.2%), but the most common leukemia was acute myeloid leukemia (37.7%). Hodgkin lymphoma was more common in female AYAs (AAIR ratio = 1.24 [1.06 to 1.45]; *P* = .006), whereas acute myeloid leukemia was equally incident (AAIR ratio = 1.09 [0.78 to 1.51]; *P* = .65). Carcinomas of the head and neck, including thyroid carcinoma, were still the most common carcinomas at 44.4%, but breast carcinomas were the second most common at 21.0%. Both thyroid and breast carcinomas were more common in female AYAs (AAIR ratios = 4.46 [3.61 to 5.56] and 69.01 [29.29 to 214.09], respectively; *P* < .001).

In the 30-39 age subgroup, the most common cancers were, again, carcinomas (n = 5,319 [64.0%]; AAIR = 29.9), lymphomas (n = 885 [10.7%]; AAIR = 4.9), and leukemias (n = 464 [5.6%]; AAIR = 2.6). Non-Hodgkin lymphoma was the predominant lymphoma (53.1%), and acute myeloid leukemia remained the most common leukemia (37.7%), followed by chronic myeloid leukemia (29.7%). Although the incidence of non-Hodgkin lymphoma was higher in male AYAs, the incidence of the myeloid leukemias did not differ between the sexes. Breast carcinoma was the most common carcinoma in this age subgroup (42.6%), and its AAIR was more than 100-fold higher in female AYAs (AAIR ratio = 103.99 [69.20 to 164.44]; *P* < .001).

**FIG 3:**
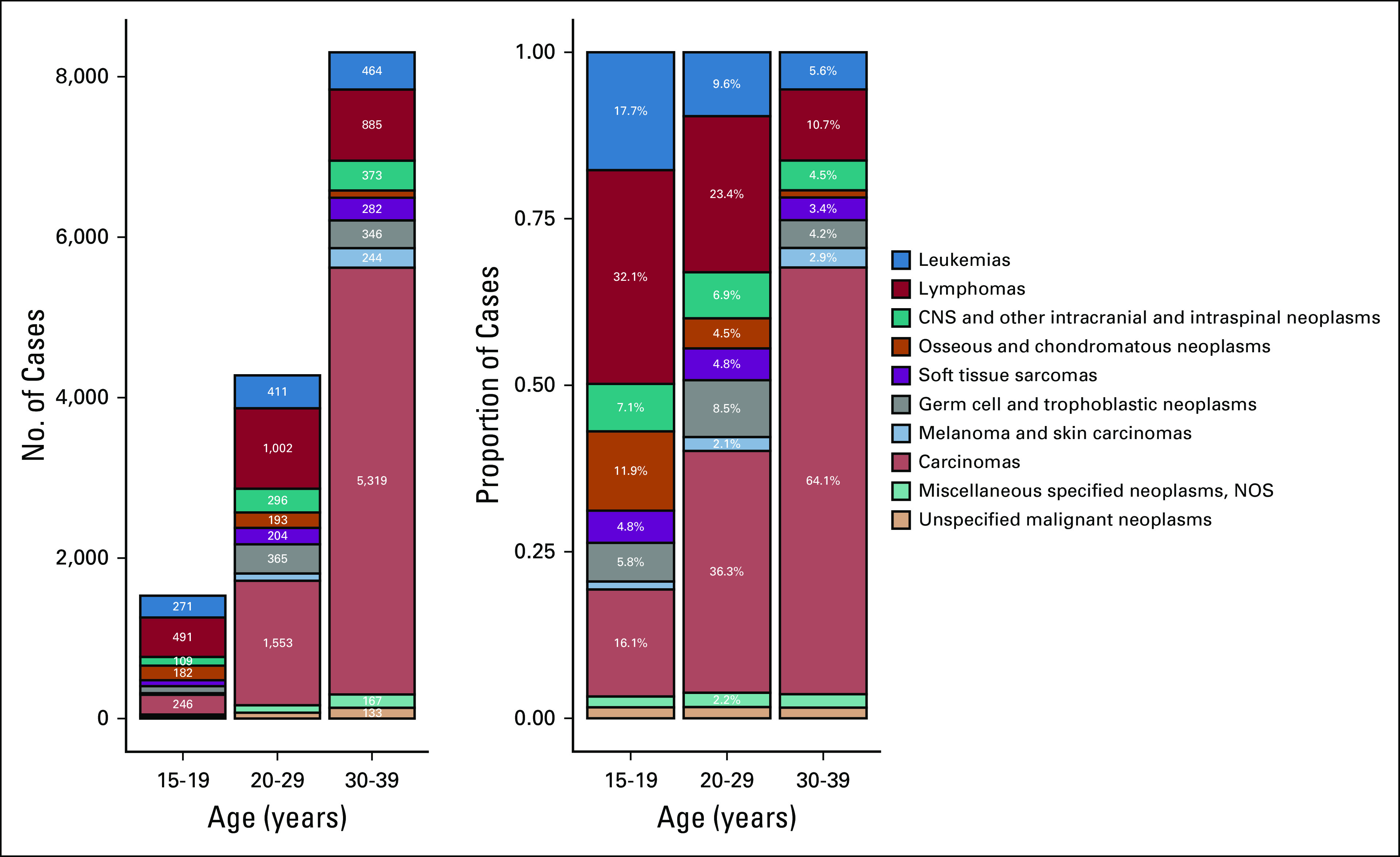
Bar plots of the number and proportion of cases of cancers diagnosed in adolescents and young adults (15-39 years) in Jordan, 2000-2017 (N = 14,115). Cases are stratified by the 15-19, 20-29, and 30-39 age subgroups. Cancers are classified according to the 10 main cancer groups of the SEER AYA site recode/WHO 2008. AYA, adolescents and young adults; NOS, not otherwise specified.

The shifting predominance of lymphomas and leukemias in the 15-19 age subgroup to carcinomas in the 30-39 age subgroup is depicted in Figure 3. Notably, osseous and chondromatous neoplasms were the fourth most common cancer in the 15-19 age subgroup but steadily accounted for a lesser proportion of cases in each successive age subgroup. On the other hand, melanoma and skin carcinomas were the least common cancer in the 15-19 age subgroup but steadily accounted for a higher proportion of cases in each successive age subgroup. Germ cell and trophoblastic neoplasms were relatively common across all three subgroups and were most incident in the 20-29 age subgroup. CNS and other intracranial and intraspinal neoplasms ranked fifth in the 15-19 and 20-29 age subgroups and fourth in the 30-39 age subgroup. Finally, soft tissue sarcomas ranked seventh in the 15-19 age subgroup and sixth in the 20-29 and 30-39 age subgroups.

We used joinpoint regression to identify changes in the AAIRs of cancer over the 18-year study period (Table 2). Of the 10 main cancer groups of the SEER AYA site recode/WHO 2008, the incidence rates of six—namely, leukemias, lymphomas, CNS and other intracranial and intraspinal neoplasms, osseous and chondromatous neoplasms, soft tissue sarcomas, and melanoma and skin carcinomas—statistically significantly decreased in a single linear trend over the full study period. On the other hand, the incidence rates of unspecified malignant neoplasms (a heterogeneous group of malignant neoplasms) statistically significantly increased in a single linear trend over the full study period. The incidence rates of germ cell and trophoblastic neoplasms and miscellaneous specified neoplasms remained constant over the full study period. Interestingly, the analysis revealed a single joinpoint for carcinomas in year 2014. Between 2000 and 2014, the yearly incidence rates of carcinomas were constant; however, between 2014 and 2017, there was a statistically significant linear downtrend.

**TABLE 2:**
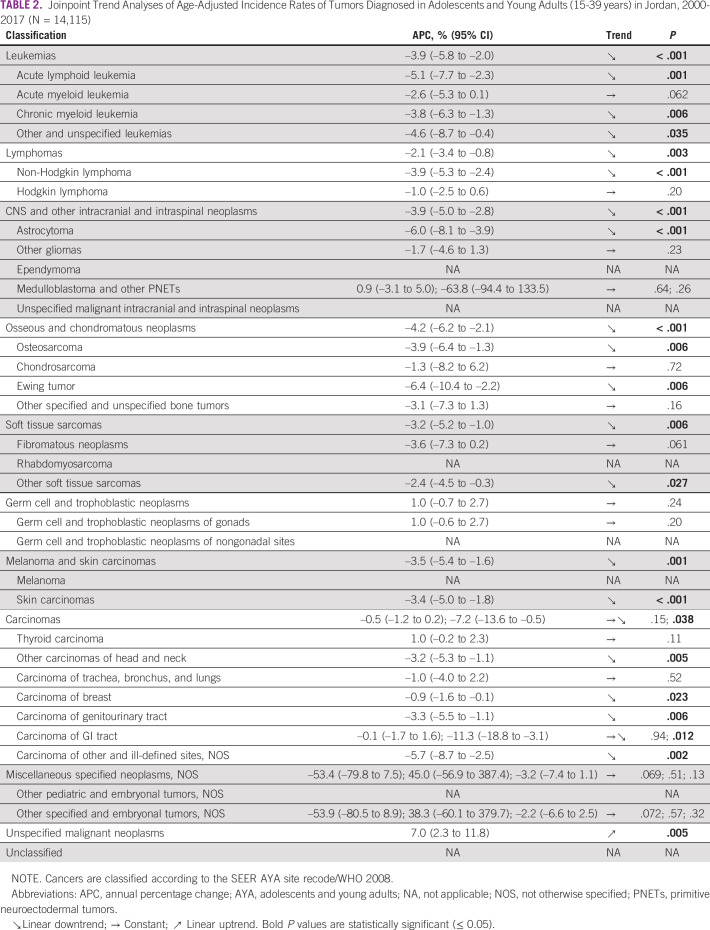
Joinpoint Trend Analyses of Age-Adjusted Incidence Rates of Tumors Diagnosed in Adolescents and Young Adults (15-39 years) in Jordan, 2000-2017 (N = 14,115)

## DISCUSSION

We accessed data from the JCR and described the incidence of cancer in AYAs in Jordan (2000-2017) using the SEER AYA site recode/WHO 2008. We found that the crude number of cases increased over time, but the AAIRs steadily decreased. We also found that the overall incidence rate of cancer was higher in female AYAs, largely because of the rate difference between the sexes in the 30-39 age subgroup. The higher incidence rates of thyroid and breast carcinomas in female AYAs largely explained this difference. In addition, we found that carcinomas, lymphomas, and leukemias were the most common cancers in AYAs. Lymphomas and leukemias accounted for almost the majority of cases in the 15-19 age subgroup, whereas carcinomas accounted for the majority of cases in the 30-39 age subgroup. Finally, we found that the incidence rates of the majority of cancers trended downward over the study period and unspecified malignant neoplasms were the only one major cancer group to trend upward.

In our study, the overall AAIR of cancer in AYAs in Jordan was 27.6 (per 100,000 population), which is comparable with the incidence rates for AYAs in other Middle Eastern countries.^4,16^ Estimates in Middle Eastern countries fall among the lower range of estimates in Asian countries, which in turn are generally lower than estimates in countries in Americas, Europe, and Oceania.^4,17,18^ In addition, many studies have reported the incidence of cancer in Jordan using data from the JCR.^7-9^ Khader et al^9^ studied the incidence of cancer in Jordan over a 14-year period (2000-2013) and reported that the overall AAIR was 126 per 100,000 population. Over the study period, the annual age-adjusted rate trended upward from 113.6 in 2000 to 142.1 in 2013. In comparison, we showed that the incidence of cancer in AYAs in Jordan trended downward from 32.3 in 2000 to 24.3 in 2017. The variation between these cancer statistics exemplifies the unique epidemiology of cancer in AYAs compared with the general population.

The distribution of cancers in our study (according to the SEER AYA site recode/WHO 2008) is generally similar to the distribution of cancers reported by other population-based registries.^2,4^ The shift in the distribution of cancers between the 15-19 and 30-39 age subgroups is also similar. However, there are some notable differences that are attributable to known population characteristics. For example, compared with our results, cutaneous melanoma is much more common in AYAs in the United States.^2^ This difference is expected because the incidence rates of cutaneous melanoma vary according to ethnicity more so than most other cancers. Indeed, cutaneous melanoma is disproportionately incident in fair-skinned White populations.^19^ Furthermore, some differences in cancer incidence are related to sexual behavior. For example, in our cohort, Kaposi sarcoma was diagnosed in seven individuals over the 18-year study period, but the disease is much more common in other countries.^2,4^ The main explanation for this difference is that Kaposi sarcoma is 20,000 times more common in people with AIDS, and the prevalence rate of AIDS in Jordan is 0.02% in the general population.^20,21^ In addition, the incidence rate of cervical carcinomas was much lower in our cohort compared with other international estimates—likely because the prevalence rate of human papillomavirus 16/18 in Jordan is relatively low.^22^

We also performed a trend analysis over the 18-year study period (2000-2017). Gupta et al^4^ studied trends in the incidence of cancer in AYAs using data from 41 countries over a 15-year period (1998-2012) and found that the annual incidence rate trended upward or remained constant in all but two countries. By contrast, we showed that the annual incidence rate in Jordan trended downward. This downtrend was also consistent across most of the major cancer site groups. We speculate that the widespread downtrend represents a decrease in the registry's access to the population at risk rather than a decrease in the burden of cancer. The number of non-Jordanian residents increased from 392,273 (7.0% of 5,580,224) in 2004 to 2,918,125 (31.5% of 9,266,575) in 2015.^11,23,24^ By contrast, the majority of our cohort were Jordanian nationals (98.3%), and 99.1% of cases in 2017 were Jordanian nationals. Therefore, non-Jordanian residents are grossly under-represented in our cohort, and the increase in the proportion of non-Jordanian residents may explain the downtrend we found. Indeed, a previous study of the burden of cancer in Syrian refugees—on the basis of data from the registry—also noted discrepancies that indicate under-reporting.^25^ Jordanian law requires that all cancer diagnoses are reported to the JCR, so non-Jordanian residents may be less likely to receive cancer care. For example, refugees constitute a large portion of the non-Jordanian population, and they may be more prone to diagnostic delay or late presentation because of poor healthcare access and utilization.^26^ Interestingly, financial limitations are the main barrier to cancer care for Syrian refugees in Jordan.^27^ On the other hand, the government bears the cost of cancer care for Jordanian nationals.^28^

Our study has several limitations. First, our cohort consists primarily of Jordanian nationals and the relative frequencies of other nationalities are disproportionately lower than population estimates. Further studies are required to investigate the incidence of cancer in AYAs from other population groups in Jordan, and the reasons for their under-representation in the national registry should be explored. Second, survival data are not well-curated so we were not able to perform survival analysis. This important research gap in survivorship must be investigated in future studies. Third, we were not able to describe other important variables, such as SEER Summary Stage 2018, because of substantial data missingness. Finally, none of the cases we studied were diagnosed postmortem because autopsies are not routinely performed in Jordan. Therefore, undiagnosed cases may partly explain the relatively low incidence of cancer in AYAs in Jordan. Further studies are required to assess the potential impact of this limitation.

In conclusion, the incidence of cancer in AYAs in Jordan is relatively low and declining. However, the absolute number of cases is increasing because this downtrend does not offset the effect of a high population growth rate; almost a 1,000 cases of cancer are now diagnosed every year, which represents a significant increase in the burden of cancer in a developing country with limited healthcare resources. Further studies are required to investigate the incidence of cancer in non-national residents.

## Figures and Tables

**TABLE A1:**
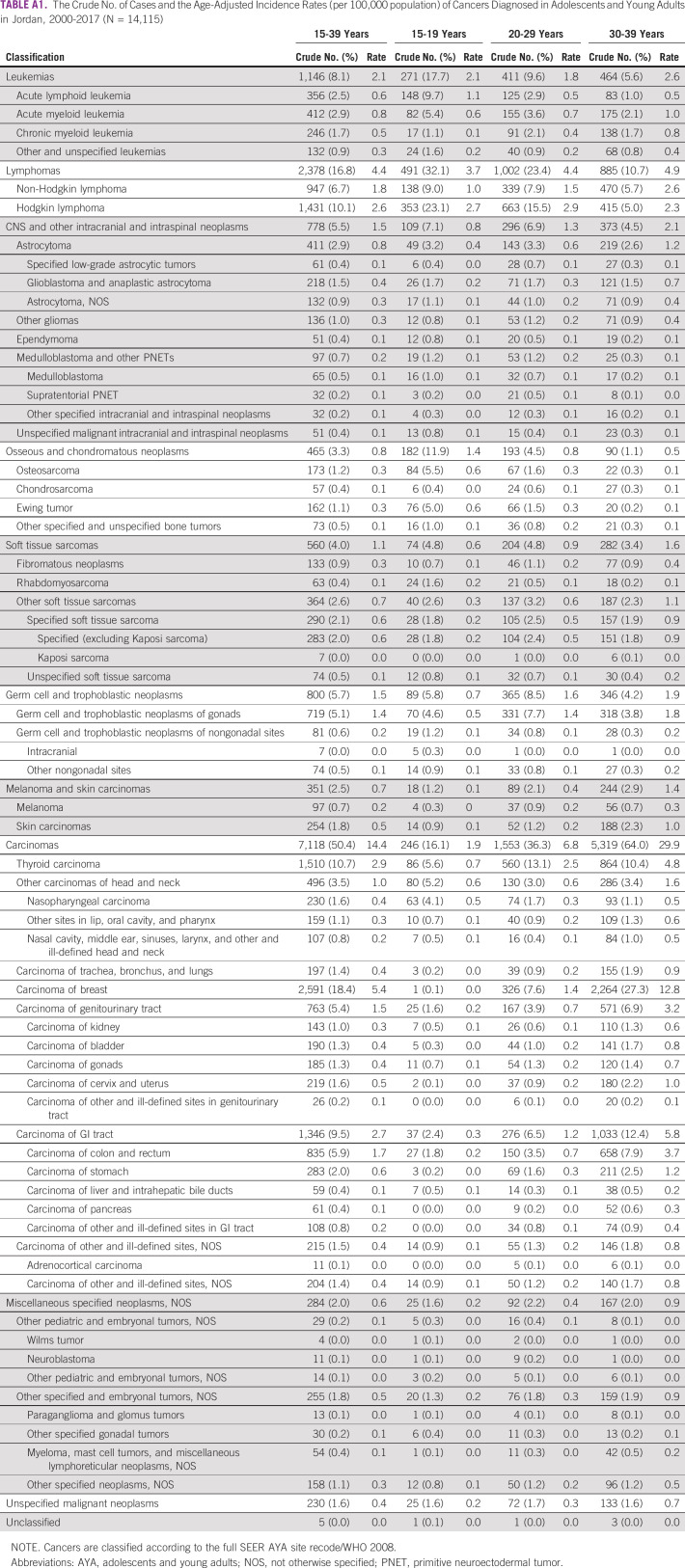
The Crude No. of Cases and the Age-Adjusted Incidence Rates (per 100,000 population) of Cancers Diagnosed in Adolescents and Young Adults in Jordan, 2000-2017 (N = 14,115)

**TABLE A2:**
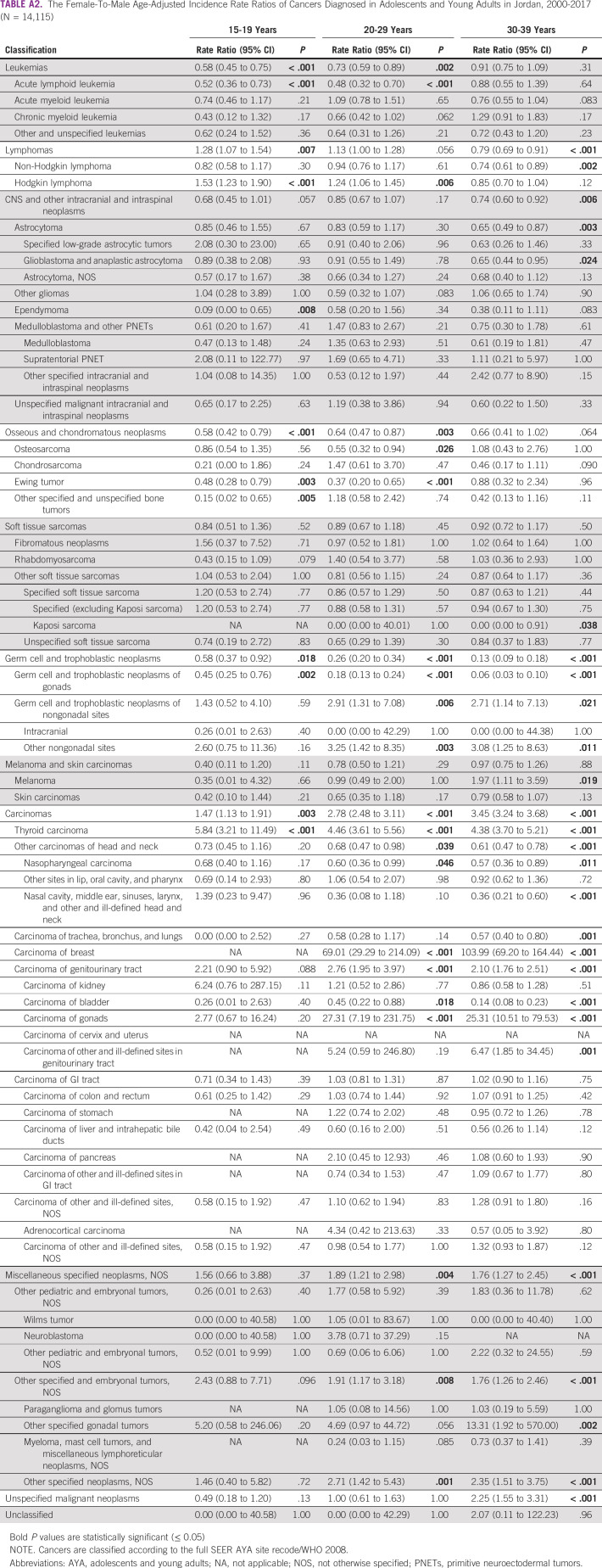
The Female-To-Male Age-Adjusted Incidence Rate Ratios of Cancers Diagnosed in Adolescents and Young Adults in Jordan, 2000-2017 (N = 14,115)

## References

[1] Zebrack B Mathews-Bradshaw B Siegel S, et al: Quality cancer care for adolescents and young adults: A position statement. J Clin Oncol 28:4862-4867, 2010 20855821 10.1200/JCO.2010.30.5417

[2] Miller KD Fidler-Benaoudia M Keegan TH, et al: Cancer statistics for adolescents and young adults, 2020. CA Cancer J Clin 70:443-459, 2020 32940362 10.3322/caac.21637

[3] Coccia PF: Overview of adolescent and young adult oncology. J Oncol Pract 15:235-237, 2019 31009282 10.1200/JOP.19.00075

[4] Gupta S Harper A Ruan Y, et al: International trends in the incidence of cancer among adolescents and young adults. J Natl Cancer Inst 112:1105-1117, 2020 32016323 10.1093/jnci/djaa007 PMC7669231

[5] Bray F Ferlay J Soerjomataram I, et al: Global cancer statistics 2018: GLOBOCAN estimates of incidence and mortality worldwide for 36 cancers in 185 countries. CA Cancer J Clin 68:394-424, 2018 30207593 10.3322/caac.21492

[6] Barr RD Holowaty EJ Birch JM: Classification schemes for tumors diagnosed in adolescents and young adults. Cancer 106:1425-1430, 2006 16544312 10.1002/cncr.21773

[7] Al-Tarawneh M Khatib S Arqub K: Cancer incidence in Jordan, 1996-2005. East Mediterr Health J 16:837-845, 2010 21469565

[8] Ismail SI Soubani M Nimri JM, et al: Cancer incidence in Jordan from 1996 to 2009--a comprehensive study. Asian Pac J Cancer Prev 14:3527-3534, 2013 23886140 10.7314/apjcp.2013.14.6.3527

[9] Khader YS Sharkas GF Arkoub KH, et al: The epidemiology and trend of cancer in Jordan, 2000-2013. J Cancer Epidemiol 2018:2937067, 2018 30416523 10.1155/2018/2937067 PMC6207872

[10] Znaor A Eser S Anton-Culver H, et al: Cancer surveillance in northern Africa, and central and western Asia: Challenges and strategies in support of developing cancer registries. Lancet Oncol 19:e85-e92, 2018 29413483 10.1016/S1470-2045(18)30019-6

[11] World Bank: World development indicators. 1960-2020. datacatalog.worldbank.org/dataset/world-development-indicators

[12] Freedman LS Al-Kayed S Qasem MB, et al: Cancer registration in the Middle East. Epidemiology 12:131-133, 2001 11138809 10.1097/00001648-200101000-00022

[13] Anton-Culver H Chang J Bray F, et al: Cancer burden in four countries of the Middle East Cancer Consortium (Cyprus; Jordan; Israel; Izmir (Turkey)) with comparison to the United States surveillance; epidemiology and end results program. Cancer Epidemiol 44:195-202, 2016 27502627 10.1016/j.canep.2016.06.004 PMC7853241

[14] Silbermann M: Manual of Standards for Cancer Registration (ed 4). 2005. www.moh.gov.cy/MOH/MOH.nsf/0/D6C382E728E5320FC22579C600265CDE/$file/MECC%20Manual%20of%20Standards%204th%20Edition%20March%202005.pdf

[15] Tiwari RC Clegg LX Zou Z: Efficient interval estimation for age-adjusted cancer rates. Stat Methods Med Res 15:547-569, 2006 17260923 10.1177/0962280206070621

[16] Bener A Ayub H Kakil R, et al: Patterns of cancer incidence among the population of Qatar: A worldwide comparative study. Asian Pac J Cancer Prev 9:19-24, 2008 18439066

[17] Stiller CA: International patterns of cancer incidence in adolescents. Cancer Treat Rev 33:631-645, 2007 17329031 10.1016/j.ctrv.2007.01.001

[18] Desandes E Stark DP: Epidemiology of adolescents and young adults with cancer in Europe. Prog Tumor Res 43:1-15, 2016 27595352 10.1159/000447037

[19] Matthews NH Li WQ Qureshi AA, et al: Epidemiology of melanoma, in Ward WH Farma JM (eds): Cutaneous Melanoma: Etiology and Therapy. Brisbane, Australia, Codon Publications, 2017, Chapter 1 29461782

[20] Cesarman E Damania B Krown SE, et al: Kaposi sarcoma. Nat Rev Dis Primers 5:9, 2019 30705286 10.1038/s41572-019-0060-9 PMC6685213

[21] Rahhal A: Evaluation of HIV/AIDS activities in Jordan, July 2018, in Refugees Operational Portal. UNHCR, 2019. data2.unhcr.org/en/documents/details/67753

[22] Bruni L Albero G Serrano B, et al: Human Papillomavirus and Related Diseases Report: Jordan. HPV Information Centre, ICO/IARC, 2019. www.hpvcentre.net/statistics/reports/JOR.pdf?t=1607960625786

[23] Department of Statistics: Population and housing census 2004, in Censuses. Department of Statistics, 2004. dosweb.dos.gov.jo/censuses/population_housing/census2015

[24] Department of Statistics: Population and housing census 2015, in Censuses. Department of Statistics, 2015. dosweb.dos.gov.jo/censuses/population_housing/census2015

[25] Mansour A Al-Omari A Sultan I: Burden of cancer among Syrian refugees in Jordan. J Glob Oncol 4:1-6, 2018 10.1200/JGO.18.00132 PMC7010435 30307806

[26] Spiegel P Khalifa A Mateen FJ: Cancer in refugees in Jordan and Syria between 2009 and 2012: Challenges and the way forward in humanitarian emergencies. Lancet Oncol 15:e290-e297, 2014 24872112 10.1016/S1470-2045(14)70067-1

[27] Al Qadire MI Alomari K: Syrian refugees in Jordan: Barriers to receiving optimal cancer care. Clin J Oncol Nurs 24:707-710, 2020 33216052 10.1188/20.CJON.707-710

[28] Abdel-Razeq H Attiga F Mansour A: Cancer care in Jordan. Hematol Oncol Stem Cell Ther 8:64-70, 2015 25732671 10.1016/j.hemonc.2015.02.001

